# High efficacy of BGD (bendamustine, gemcitabine, and dexamethasone) in relapsed/refractory Hodgkin Lymphoma

**DOI:** 10.1007/s00277-021-04448-5

**Published:** 2021-02-24

**Authors:** Ryszard Swoboda, Sebastian Giebel, Wanda Knopińska-Posłuszny, Ewa Chmielowska, Joanna Drozd-Sokołowska, Ewa Paszkiewicz-Kozik, Waldemar Kulikowski, Michał Taszner, Włodzimierz Mendrek, Jacek Najda, Tomasz Czerw, Magdalena Olszewska-Szopa, Anna Czyż, Agnieszka Giza, Wojciech Spychałowicz, Edyta Subocz, Paweł Szwedyk, Aleksandra Krzywon, Agata Wilk, Jan Maciej Zaucha

**Affiliations:** 1 Department of Bone Marrow Transplantation and Oncohematology, Maria Sklodowska-Curie National Research Institute of Oncology, Gliwice branch, Gliwice, Poland; 2 Department of Hematology and Bone Marrow Transplantation, Pomeranian Hospitals, Gdynia, Poland; 3 Oncologic Hospital, Tomaszów Mazowiecki, Poland; 4 Department of Oncology, Oncology Centre, Bydgoszcz, Poland; 5 Department of Hematology, Oncology and Internal Diseases, Medical University of Warsaw, Warsaw, Poland; 6 Department of Lymphoid Malignancies, Maria Sklodowska-Curie National Research Institute of Oncology, Warsaw branch, Warsaw, Poland; 7 Department of Hematology, Independent Public Health Care Ministry of the Interior of Warmia and Mazury Oncology Center, Olsztyn, Poland; 8 Department of Hematology and Transplantology, Medical University of Gdańsk, Gdańsk, Poland; 9 Department of Hematology, Blood Neoplasms and Bone Marrow Transplantation, Wrocław Medical University, Wrocław, Poland; 10 Department of Hematology, Jagiellonian University, Krakow, Poland; 11 Internal Medicine and Oncology Clinic, Silesian Medical University, Katowice, Poland; 12 Department of Hematology, Military Institute of Medicine, Warsaw, Poland; 13 Department of Hematology, Ludwik Rydygier Hospital, Krakow, Poland; 14 Department of Biostatistics and Bioinformatics, Maria Sklodowska-Curie National Research Institute of Oncology, Gliwice Branch, Gliwice, Poland

**Keywords:** Hodgkin lymphoma, Chemotherapy, Treatment, Salvage

## Abstract

The optimal salvage therapy in relapsed/refractory Hodgkin lymphoma (R/R HL) has not been defined so far. The goal of this multicenter retrospective study was to evaluate efficacy and safety of BGD (bendamustine, gemcitabine, dexamethasone) as a second or subsequent line of therapy in classical R/R HL. We have evaluated 92 consecutive R/R HL patients treated with BGD. Median age was 34.5 (19–82) years. Fifty-eight patients (63%) had received 2 or more lines of chemotherapy, 32 patients (34.8%) radiotherapy, and 21 patients (22.8%) an autologous hematopoietic stem cell transplantation (autoHCT). Forty-four patients (47.8%) were resistant to first line of chemotherapy. BGD therapy consisted of bendamustine 90 mg/m^2^ on days 1 and 2, gemcitabine 800 mg/m^2^ on days 1 and 4, dexamethasone 40 mg on days 1–4. Median number of BGD cycles was 4 (2–7). The following adverse events ≥ 3 grade were noted: neutropenia (22.8%), thrombocytopenia (20.7%), anemia (15.2%), infections (10.9%), AST/ALT increase (2.2%), and skin rush (1.1%). After BGD therapy, 51 (55.4%) patients achieved complete remission, 23 (25%)—partial response, 7 (7.6%)—stable disease, and 11 (12%) patients experienced progression disease. AutoHCT was conducted in 42 (45.7%) patients after BGD therapy, and allogeneic HCT (alloHCT) in 16 (17.4%) patients. Median progression-free survival was 21 months. BGD is a highly effective, well-tolerated salvage regimen for patients with R/R HL, providing an excellent bridge to auto- or alloHCT.

## Introduction

Hodgkin lymphoma (HL), accounting for approximately 10% of all lymphomas, is one of the most curable malignancies. However, up to 30% of patients do not respond to the first-line therapy or relapse after initial response [1]. For patients with relapsed or refractory disease, salvage chemotherapy followed by high-dose chemotherapy with autologous hematopoietic stem cell transplantation (autoHCT) is still the treatment of choice [2, 3]. The long-term cure can be obtained in up to 80% of patients provided a complete metabolic remission (CMR) is achieved before transplant [4]. Consequently, the optimal salvage regimen should be highly effective but also should have a high mobilization rate. However, the standard for salvage chemotherapy before autoHCT is still not established. The most commonly used are platinum-based ICE (ifosfamide, carboplatin, etoposide); DHAP (dexamethasone, cytarabine, cisplatin); ESHAP (etoposide, methylprednisolone, cytarabine, cisplatin); and gemcitabine-based GDP (gemcitabine, dexamethasone, cisplatin), GVD (gemcitabine, vinorelbine, liposomal doxorubicin), IGEV (ifosfamide, gemcitabine, vinorelbine), and GCD (gemcitabine, carboplatin, dexamethasone) combination chemotherapy with response rates ranging between 54 and 88% and mobilization rate of 86–100% [5–10]. Due to rather low rate of complete responses (CRs) achieved with the most frequently used salvage regimens, there is the persisting need to develop new salvage regimens especially in the second-line treatment. Recently, bendamustine both in monotherapy and in combination with other drugs was shown to induce high response rates with an acceptable toxicity profile in third or more line in patients with relapsed/refractory (R/R) HL [11–13]. Experience with bendamustine in the second line is very limited. Santoro et al. modified their original IGEV protocol substituting ifosfamide with bendamustine achieving a very high efficacy as a second-line therapy in patients with R/R HL [9, 14]. The Polish Lymphoma Research Group (PLRG) proposed replacement of vinorelbine with dexamethasone (bendamustine, gemcitabine, dexamethasone; BGD regimen) and is carrying out a prospective, multicenter study in patients with progressive disease during or after ABVD treatment [15] based on the very good preliminary results obtained with BGD in heavily pretreated R/R HL patients [16]. Here we report long-term outcome of these patients enrolled to the multicenter retrospective PLRG study aiming at evaluating the efficacy and toxicity of BGD in a real-life setting.

## Methods

### Study design

We retrospectively reviewed the data of all patients aged ≥ 18 years with R/R HL who were treated with BGD regimen between April 2012 and December 2018 in 15 centers allied within the PLRG. Primary refractory HL was defined as no CMR achieved after the first line or if progression occurred within 3 months after completion of the first-line chemotherapy*.* In patients with relapsed HL, the disease reappeared later after achieving CMR. Patients’ records were reviewed to obtain patient characteristics at diagnosis and the start of BGD treatment, including clinical stage according to the Lugano system, presence of B symptoms, extranodal site involvement, and bulky disease.

### Treatment and response criteria

The dosage and administration schedule of BGD is shown in Table 1. The interim imaging assessment was performed after second or third cycle of BGD in 86 patients (93.5%). Patients could continue BGD treatment up to 4 cycles or longer at the discretion of a treating physician. The metabolic response to BGD treatment at the end of the therapy was assessed according to the Lugano treatment response criteria using 18F-fluorodeoxyglucose (18F-FDG) positron emission tomography/computed tomography (PET/CT) [17]. The 18F-FDG uptake less than in the liver was defined as CMR. The higher 18F-FDG uptake, but decreased from baseline, was defined as partial metabolic response (PMR). In case of no significant change in 18F-FDG uptake from baseline or new FDG-avid foci, stable or progressive disease (SD or PD) were diagnosed, respectively. The overall response rate (ORR) was defined as the sum of CMR and PMR.

**Table 1:** BGD regimen repeated every 4 weeks

Drug	Dose	Route of administration^a^	Day of administration
Bendamustine	90 mg/m^2^	*i.v.* 60 min	1 and 2
Gemcitabine	800 mg/m^2^	*i.v.* 30 min	1 and 4
Dexamethasone	40 mg	*i.v.* or *p.o.*	1, 2, 3, and 4

^a^*i.v.*, intravenous; *p.o.*, per os

### Study end-points and statistical analysis

Primary end-point was the percentage of CMR and ORR whereas progression-free survival (PFS) and overall survival (OS) as well as adverse events (AEs) were secondary end-points. PFS was defined as the time from start of BGD treatment to the date of documented disease progression, death from any cause, or start of new anticancer therapy. The patients at the time of autoHCT or allogeneic hematopoietic stem cell transplantation (alloHCT) were censored for PFS since transplants were not considered a new anticancer therapy. OS was defined as the time from the first BGD administration to death for any reason. AEs were evaluated using Common Terminology Criteria for Adverse Events (CTCAE), version 4.0 [18]. PFS and OS were estimated using the Kaplan-Meier method. Ninety-five percent confidence intervals (CIs) for the survival curves were calculated for chosen times. The log-rank test was performed to compare survival curves between groups. Two-sided *P* values ≤ 0.05 were considered statistically significant. The statistics were performed descriptively. Statistical analysis was performed using Statistica software (version 13.1, Tulsa, OK, USA) and R statistical software package version 4.0.1. (R Foundation for Statistical Computing, http://www.r-project.org).

## Results

### Patients’ characteristics

The median age of 92 analyzed patients was 34.5 (range from 19 to 82 years) years with 9 patients at the age or above 60 years. More than 60% of patients were in advanced stages of HL at the time of diagnosis (III and IV according to the Lugano classification). Median number of prior chemotherapy lines was 2 (range from 1 to 6 lines). Thirty-four patients (37%) had previously been treated with only one line of chemotherapy. ABVD was the first-line therapy in 80.4% cases. Forty-four patients (47.8%) were primary refractory to the first-line chemotherapy. Almost 35% of patients had received radiotherapy prior to BGD treatment. Twenty-one patients (22.8%) underwent autoHCT, and three patients (3.3%) underwent alloHCT prior to BGD treatment. The median number of BGD cycles was 4 (range from 2 to 7 cycles). Demographic and clinical data are shown in Table 2.

**Table 2:** Demographics and clinical characteristics of 92 subjects

Characteristic	No.	%
Age, years		
- Median (range)	34.5 (19–82)	
- ≥ 60 years	9	9.7
Sex		
- Male	44	47.8
- Female	48	52.2
Lugano classification at diagnosis/before BGD^a^		
- I	0/3	0/3.3
- II	33/38	35.9/41.3
- III	25/9	27.2/9.8
- IV	34/42	37/45.7
B-symptoms at diagnosis/before BGD^a^	67/27	72.9/29.3
Extranodal site involvement at diagnosis/before BGD^a^	32/39	34.8/42.4
Bulky disease at diagnosis/before BGD^a^	32/10	34.8/10.9
First-line treatment		
ABVD^b^	74	80.4
BEACOPP^c^	13	14.1
Other^d^	5	5.4
No. of previous treatment lines		
- 1.	34	37
- 2.	26	28.3
- 3.	16	17.4
- 4.	9	9.8
- 5.	3	3.3
- 6.	4	4.3
Primary refractory to 1st-line treatment	44	47.8
Patents with late (> 12 months) relapse	20	21.7
No. of BGD^a^ courses		
- Median (range)	4 (2–7)	
Radiotherapy prior to BGD^a^	32	34.8
Prior autoHCT^e^	21	22.8
Prior alloHCT^f^	3	3.3

^a^*BGD*, bendamustine, gemcitabine, dexamethasone

^b^*ABVD*, adriamycin, bleomycin, vinblastine, dacarbazine

^c^*BEACOPP*, bleomycin, cyclophosphamide, doxorubicin, etoposide, prednisone, procarbazine, vincristine

^d^Other: *PVAG*, prednisone, vincristine, adriamycin, gemcitabine; *OEPA/COPDAC*, vincristine, etoposide, prednisone, doxorubicin/ cyclophosphamide, vincristine, prednisone, dacarbazine

^e^*autoHCT*, autologous hematopoietic cell transplantation

^f^*alloHCT*, allogeneic hematopoietic cell transplantation

### Efficacy of BGD

**Fig. 1:**
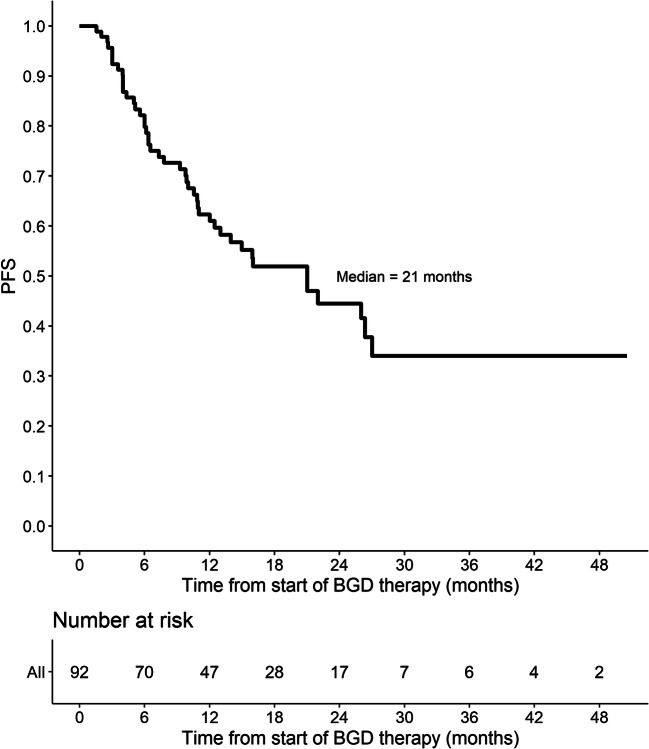
Progression-free survival (PFS) for the entire population

**Fig. 2:**
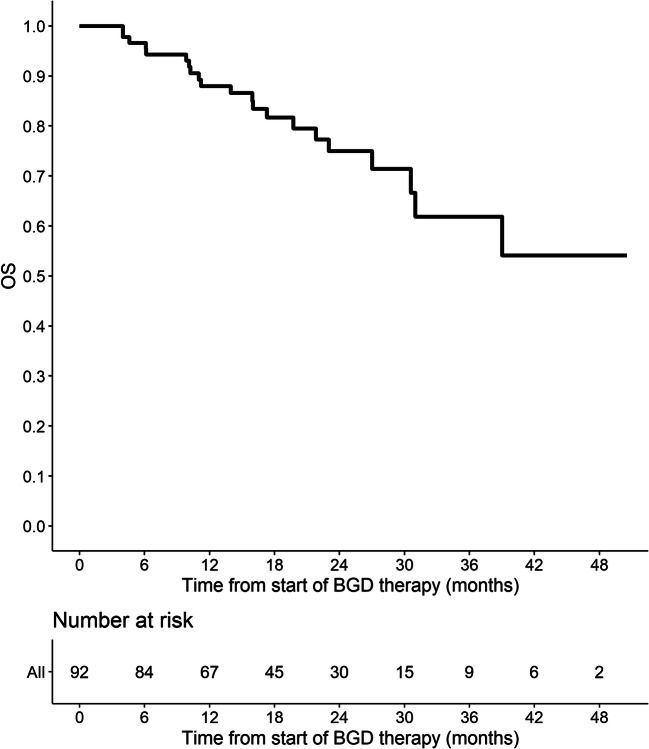
Overall survival (OS) for the entire population

**Fig. 3:**
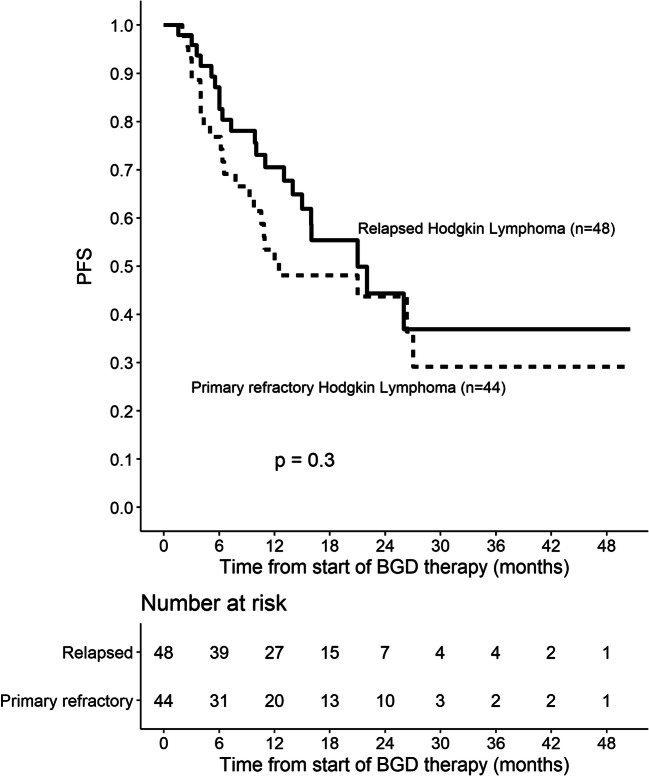
Progression-free survival (PFS) for the subjects with relapsed or primary refractory Hodgkin lymphoma (HL)

**Fig. 4:**
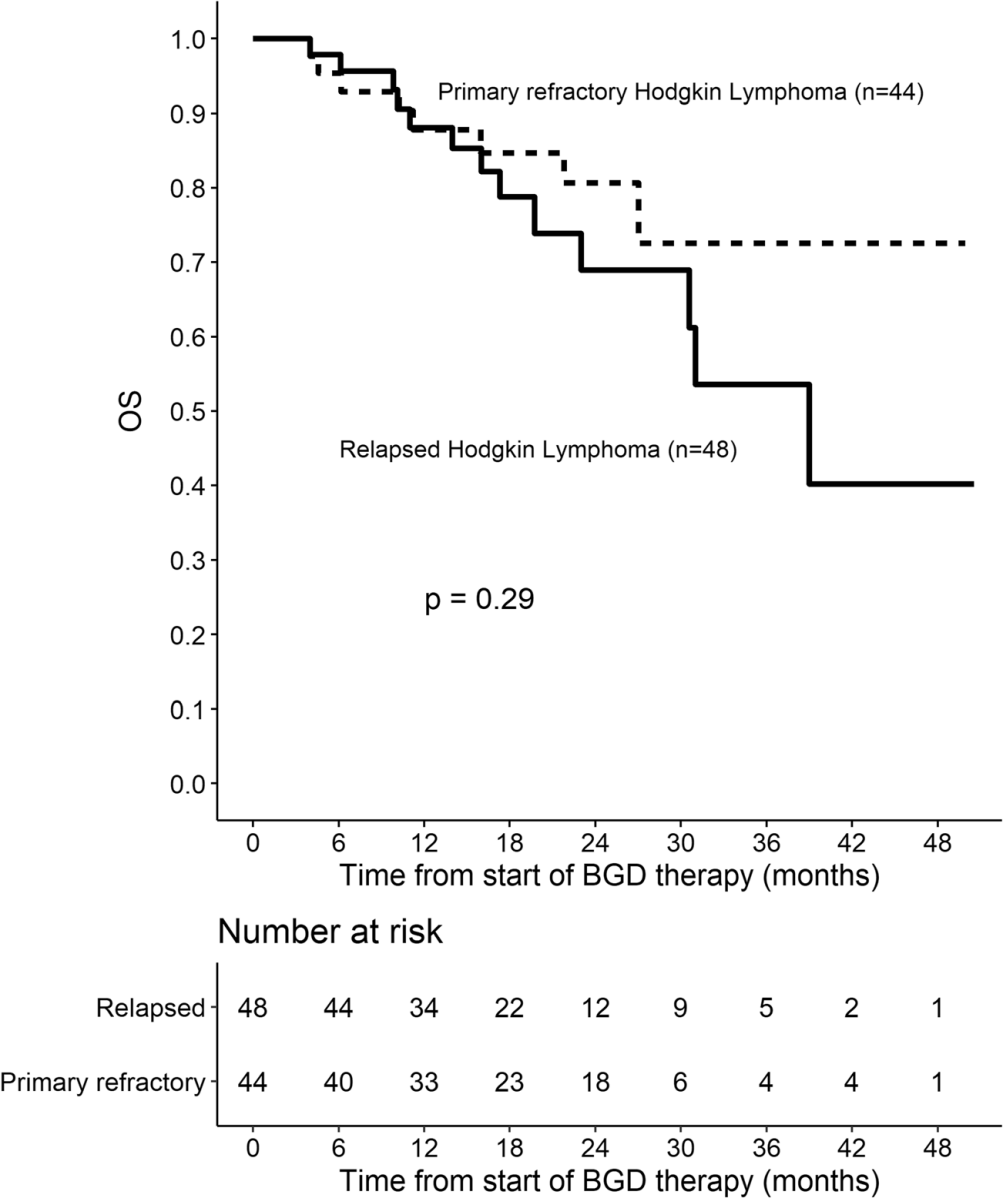
Overall survival (OS) for the subjects with relapsed or primary refractory Hodgkin lymphoma (HL)

**Fig. 5:**
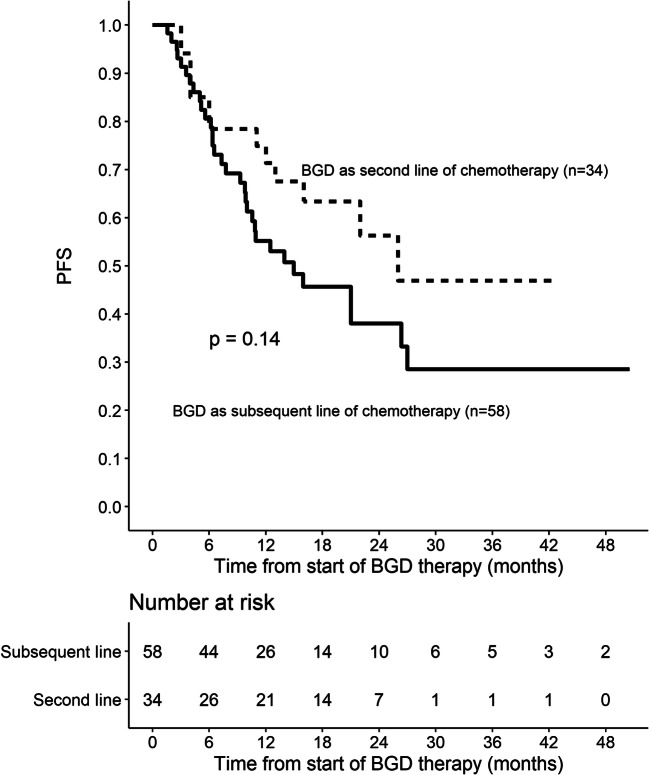
Progression-free survival (PFS) for patients treated with BGD as second or subsequent line of chemotherapy

**Fig. 6:**
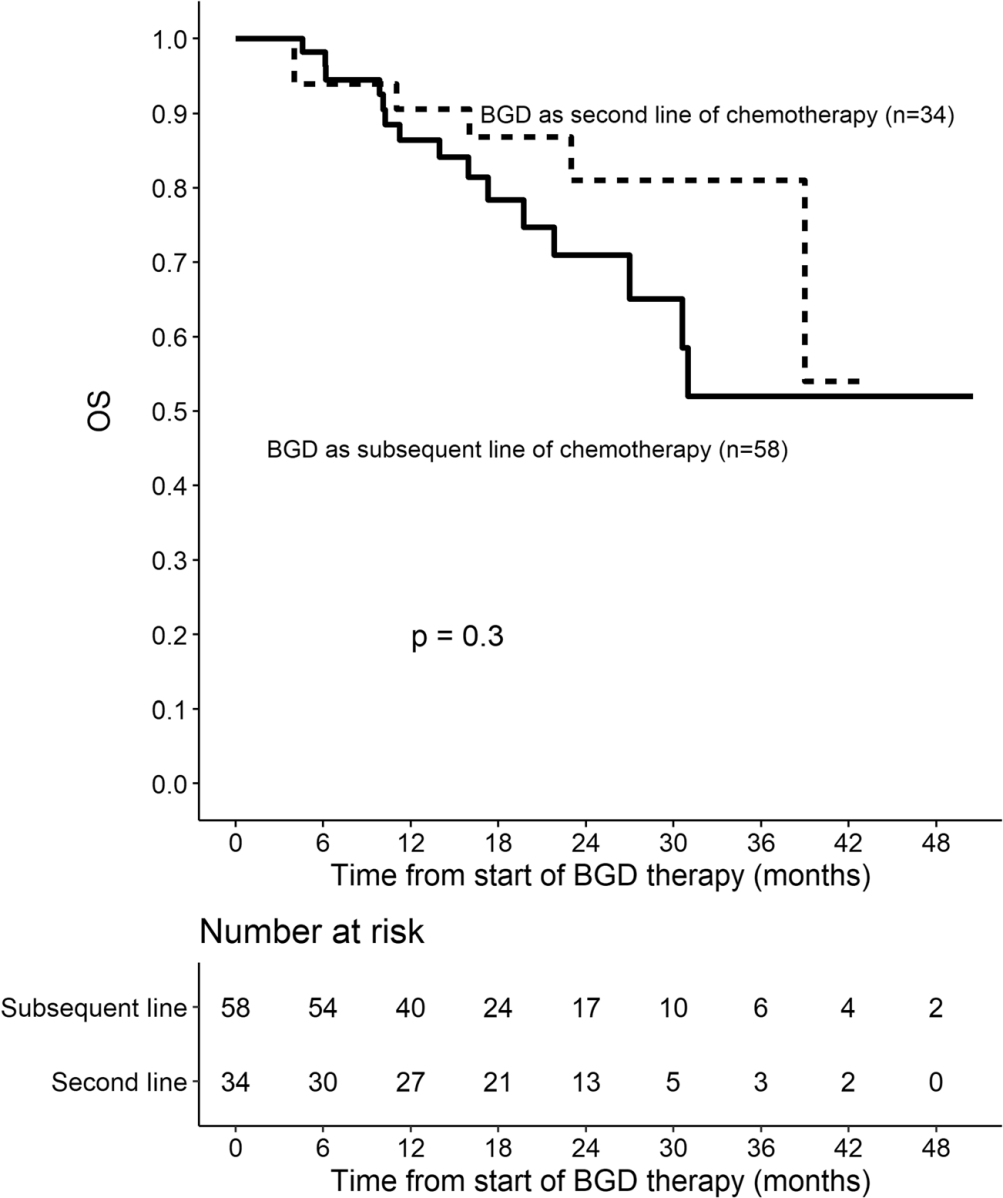
Overall survival (OS) for patients treated with BGD as second or subsequent line of chemotherapy

**Fig. 7:**
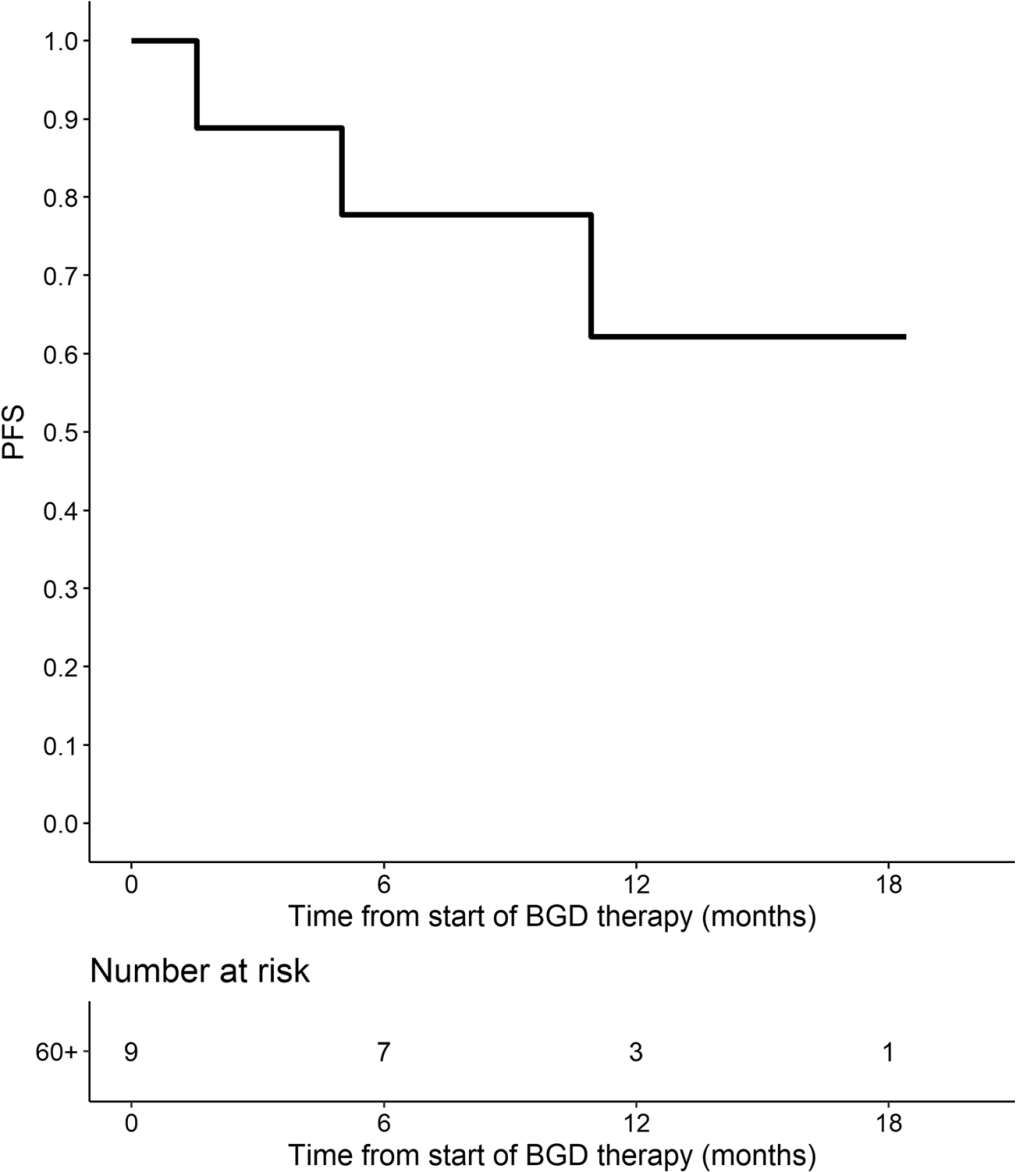
Progression-free survival (PFS) for the subgroup of elderly patients (≥ 60 years)

**Fig. 8:**
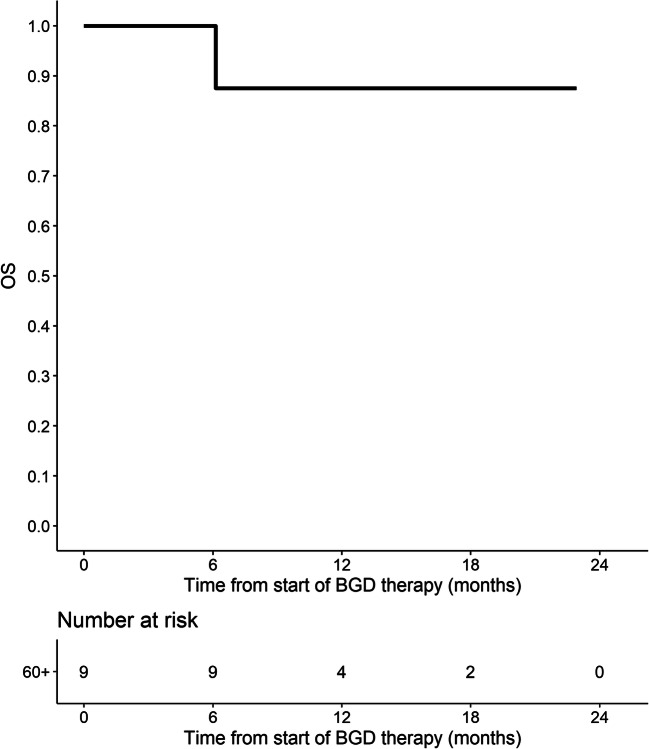
Overall survival (OS) for the subgroup of elderly patients (≥ 60 years)

Out of 86 patients who were assessed by interim PET/CT after 2 or 3 cycles of BGD, 67 (77.9%) patients achieved overall response, including 33 (38.4%) CMRs. At the end of BGD therapy, ORR and CMR rate increased to 80.4% and 55.4%, respectively. Eleven patients (12%) experienced disease progression, and seven patients (7.6%) had stable disease. Among 34 patients who achieved PMR in the interim imaging test, 14 (41.2%) patients achieved CMR at the end of BGD treatment. In the subgroup of patients treated with BGD only as a second line of chemotherapy (*n* = 34), ORR was 79.4% while CMR rate was 64.7%. Out of 44 patients being refractory to the first line of chemotherapy, 30 (68.2%) patients achieved overall response and 34.1% achieved CMR. Among the whole study population, five patients were refractory to BEACOPP given as the first line of chemotherapy. All these patients achieved response and two of them (40%) achieved CMR after BGD. The autoHCT was successfully performed after BGD therapy in 42 (45.7%) patients. Furthermore, 16 (17.4%) patients underwent alloHCT after BGD therapy (Table 3). Among 21 patients treated with autoHCT before BGD therapy, 18 (85.7%) achieved response, 11 (52.4%) CMR, and 9 (42.9%) patients proceeded to alloHCT. PFS and OS are shown as Kaplan-Meier curves in Figs. 1 and 2. With a median follow-up of 18 months (range from 3 to 51 months), the median PFS for the overall population was 21 months while the median OS was not reached. PFS and OS rates at 2 years were 44.5% (95%CI: 33.7–58.8) and 75% (95%CI: 64.8–86.7), respectively. There were no differences between patients with relapsed or primary refractory HL in terms of PFS and OS (Figs. 3 and 4, *P* > 0.05). Similarly, no statistical differences in PFS and OS were noted between patients in whom BGD was used after first line or subsequent line of chemotherapy (Figs. 5 and 6, *P* > 0.05). Of note, most of the patients (80%) who responded to BGD maintain their response for at least 6 months (Fig. 1). In a subgroup of 9 patients ≥ 60 years old, 5 (55.6%) patients achieved CMR, 2 (22.2%)—PMR (ORR 77.8%), and 2 patients progressed during BGD treatment. PFS at 18 months was 62.2% (95%CI: 35.5–100) and OS at 23 months was 87.5% (95%CI: 67.3–100) (Figs. 7 and 8).

**Table 3:** Response to BGD therapy

	No.	% (95%CI)
Interim (after 2–3 courses)	*n* = 86	
- PMD^a^	6	7 (1.6–12.4)
- SMD^b^	13	15.1 (7.5–22.7)
- PMR^c^	34	39.5 (29.2–49.9)
- CMR^d^	33	38.4 (28.1–48.6)
- ORR^e^	67	77.9 (69.1–86.7)
End of BGD^f^ therapy	*n* = 92	
- PMD^a^	11	12 (5.3–18.6)
- SMD^b^	7	7.6 (2.2–13.0)
- PMR^c^	23	25 (16.2–33.8)
- CMR^d^	51	55.4 (45.3–65.6)
- ORR^e^	74	80.4 (2.3–88.5)
AutoHCT^g^ after BGD therapy		
- Yes	42	45.7
- No	50	54.3
AlloHCT^h^ after BGD therapy		
- Yes	16	17.4
- No	76	82.6

^a^*PMD*, progressive metabolic disease

^b^*SMD*, stable metabolic disease

^c^*PMR*, partial metabolic response

^d^*CMR*, complete metabolic response

^e^*ORR*, overall response rate

^f^*BGD*, bendamustine, gemcitabine, dexamethasone

^g^*autoHCT*, autologous hematopoietic cell transplantation

^h^*alloHCT*, allogeneic hematopoietic cell transplantation

### Toxicity of BGD

The treatment-related AEs, mainly grade 1 or 2, were observed in 64 (69.6%) patients. Among grade ≥ 3 hematological toxicities, anemia was reported in 15.2% cases, thrombocytopenia in 20.7%, and neutropenia in 22.8% individuals. Severe non-hematological toxicities included infections (10.9%), alanine and/or aspartate aminotransferase (ALT or AST) increase (2.2%), and skin rush (1.1%). We recorded one death from unknown reasons during BGD therapy. All AEs reported during BGD therapy are shown in Table 4. In the subgroup of elderly patients (≥ 60 years), anemia and thrombocytopenia of grade 3 or 4 occurred in 2 (22.2%) patients, while neutropenia was found in 1 (11.1%) patient. Moreover, 2 (22.2%) patients experienced grade ≥ 3 infection, and 1 (11.1%) grade 3 skin rash in this subgroup.

**Table 4:** Toxicity of BGD therapy

Toxicity	Grade 1–2 No. (%)	Grade 3–4 No. (%)
Hematological:		
- Anemia	37 (40.2)	14 (15.2)
- Thrombocytopenia	25 (27.2)	19 (20.7)
- Neutropenia	19 (20.7)	21 (22.8)
Non-hematological:		
- Infection	20 (21.7)	10 (10.9)
- ALT/AST^a^ increase	0	2 (2.2)
- Skin rush	7 (7.6)	1 (1.1)
- Fatigue	4 (4.3)	0
- Diarrhea	1 (1.1)	0
- Thrombotic events	2 (2.2)	0
- Guillain-Barré syndrome	1 (1.1)	0
- Autoimmune thyroiditis	1 (1.1)	0
Death from unknown reasons	1 (1.1)	

^a^Alanine/aspartate aminotransferase

## Discussion

Despite the significant progress in the management of patients with HL, relapsed and refractory disease constitutes a big challenge. According to the current treatment standards, high-dose chemotherapy followed by autoHCT has to be regarded as treatment of choice in the first relapse or primary progressive disease. However, even in the era of new agents such as brentuximab vedotin (BV) or immune checkpoint inhibitors, there are no accepted standards for salvage treatment before autoHCT. In the PLRG allied centers BGD was used since 2012 when the first cases of successful outcome of heavily pretreated patients were reported [16]. We had started to use BGD since both bendamustine and gemcitabine were effective in monotherapy as well as in combination with other agents.

Gemcitabine, a cytidine analog, was assessed in several studies. Kuruvilla et al. showed satisfactory (62%) response rate to GDP (gemcitabine, dexamethasone, and cisplatin) as a second-line therapy in patients with R/R HL [19]. Gemcitabine and vinerolbine combination showed ORR of 72% and 35% of CR [20]. In similar population, four-agent combination of gemcitabine with ifosfamide, vinorelbine, and prednisolone (IGEV) or double-agent schema of vinorelbine and pegylated liposomal doxorubicin (GVD) resulted in ORR of 81.3% and 70%, respectively [8, 9]. Bendamustine, another component of BGD, is a molecule containing the purine analog and the alkylating group. It was assessed in a phase II trial in heavily pretreated patients with HL (including those with relapse after auto- or alloHCT) at the dose of 120 mg/m^2^ and resulted in the intent-to-treat ORR of 53%, with CRs of 33% [11].

The combination of bendamustine with gemcitabine and vinorelbine (BeGEV) was first assessed in a multicenter phase II study by Santoro et al. [14]. The authors have demonstrated high efficacy of BeGEV as a second-line treatment in autoHCT-eligible patients (ORR 83%, CR 75%). In our retrospective analysis, ORR of BGD was similar to BeGEV although patient characteristics with regard to age and number of prior treatment regimens differ [14]. Moreover, almost one-fourth of patients in our study had been treated using autoHCT. This also most likely explains slightly higher rate of hematological toxicities at grade 3 or 4 in comparison to BeGEV. In contrast, severe non-hematological AEs occurred at similar incidence rates. We did not observe increased pulmonary toxicity which was reported by Cohen et al. in his cohort of patients. This might be due to the use of high dose of steroids (dexamethasone) in our regimen [21]. However, in spite of high efficacy of BGD in relation to ORR and CMR rate, estimated PFS in our study seems to be rather short (21 months). Most likely, this is a consequence of the large group of heavily pretreated patients (up to 6 previous line of therapy) including those after auto- and alloHCT and the subgroup of elderly patients in this cohort.

DHAP and ICE are among of the most commonly used salvage chemotherapy regimens in HL. Similar efficacy of these regimens as the second-line treatment was shown resulting in ORR of 88% for both, while CRs of 21% for DHAP and of 26.2% for ICE [5, 6]. Despite more heavily pretreated patients in our study, we report comparable ORR (80.4%) and much higher CMR rate (55.4%). Moreover, the difference in relation to CR rate increases in sub-analysis restricted to patients treated with BGD as the second line of chemotherapy (64.7%). The optimal number of BGD cycles is four, since about 40% of patients improved their response at the end of treatment compared to the interim assessment.

Most patients (80.4%) in this study were treated with ABVD as the first line of chemotherapy. Out of 13 patients being initially treated with BEACOPP, 5 (38.5%) patients were refractory to this regimen and all these patients achieved ORR after BGD therapy with CMR rate of 40%. In view of the fact that there are not many effective treatment options to overcome the BEACOPP refractoriness, this finding seems very interesting and encouraging for further studies.

In recent years, there have been many new agents evaluated for patients with R/R HL such as BV and immune checkpoint inhibitors. Brentuximab vedotin—an anti-CD30 antibody conjugated to antimicrotubule agent, monomethyl auristatin E—showed ORR of 75% and CR rate of 34% in heavily pretreated patients with HL after autoHCT [22]. Furthermore, BV showed similar efficacy as a second-line therapy in R/R HL (ORR 68%, CR 35%), and 89% of the subjects were able to proceed to autoHCT [23]. Addition of bendamustine to BV seems to increase its efficacy [12, 13]. However, in comparison to BGD regimen, BV does not appear to be superior both in monotherapy and in combination with bendamustine. Immune checkpoint inhibitors, mainly antibodies against programmed cell death protein 1 (PD-1) such as nivolumab or pembrolizumab, showed high efficacy as a salvage therapy for patients with R/R HL [24–27]. Ansell et al. reported ORR 87% in the heavily pretreated HL patients although CR rate was relatively low (17%) compared to BGD [24]. The authors reported similar incidence of AEs of any grade (78% vs. 69.6%) but relatively fewer cases of grade ≥ 3 AEs (22% vs. 38%) during nivolumab therapy when compared to BGD therapy [24].

The high efficacy of BGD in patients after failure of autoHCT is worth noting. This is a very challenging group, in which effective chemotherapy followed by alloHCT is the only option to achieve long-lasting remission. In this population checkpoint inhibitors, nivolumab and pembrolizumab resulted in high ORR rate (69% and 73%, respectively). However, once again, CR rates (16% and 14%, respectively) appear lower compared to BGD in our study (55.4%) and there are still some concerns about the increased incidence of immune complications after alloHCT in patients treated with immune checkpoint inhibitors before transplant [25, 26, 28, 29].

Another interesting finding of this study refers to elderly HL patients. We showed that in the patients at or above the age of 60 years BGD regimen has a similar efficacy (ORR 77.8%, CMR 55.6%) than in younger patients. Moreover, BGD-related toxicity seems to be acceptable in this subgroup.

Relatively small number of subjects included in the analysis as well as its retrospective nature and the lack of metabolic assessment in all patients are the major limitations of this study. Another important limitation of our study is a long accrual time. However, during this period, most of our patients were treated uniformly since novel therapies were not available due to reimbursement hurdles. Addition limitation may pose a large number of accruing centers. However, most of PLRG allied centers are reference centers with good-quality data management which somehow mitigate this flaw. However, our data clearly indicate that BGD which has a high rate of obtaining ORR and a relatively good time in response maintaining (5–6 months in 80% of patients) and an acceptable toxicity profile seems to provide a very good option for R/R HL patients even after BEACOPP first-line chemotherapy. In several patients, it opened a window to perform either autologous or allogeneic transplantation. Despite the fact that in recent years new treatment options, including BV and checkpoint inhibitors, showed promising results in treatment of patients with R/R HL, neither of them as yet is formally approved in the second-line treatment for HL. In addition, although pharmaco-economy was not a subject of this study, we speculate that BGD treatment may be a relatively cheap option. Therefore, we conclude that the BGD should be considered a viable option for patients with R/R HL and may serve as a bridge for individuals being candidates for auto- or alloHCT.

## Data Availability

Not applicable
