# Correction to: High efficacy of BGD (bendamustine, gemcitabine, and dexamethasone) in relapsed/refractory Hodgkin lymphoma

**DOI:** 10.1007/s00277-021-04503-1

**Published:** 2021-03-24

**Authors:** Ryszard Swoboda, Sebastian Giebel, Wanda Knopińska-Posłuszny, Ewa Chmielowska, Joanna Drozd-Sokołowska, Ewa Paszkiewicz-Kozik, Waldemar Kulikowski, Michał Taszner, Włodzimierz Mendrek, Jacek Najda, Tomasz Czerw, Magdalena Olszewska-Szopa, Anna Czyż, Agnieszka Giza, Wojciech Spychałowicz, Edyta Subocz, Paweł Szwedyk, Aleksandra Krzywon, Agata Wilk, Jan Maciej Zaucha

**Affiliations:** 1 Department of Bone Marrow Transplantation and Oncohematology, Maria Sklodowska-Curie National Research Institute of Oncology, Gliwice branch, Gliwice, Poland; 2 Department of Hematology and Bone Marrow Transplantation, Pomeranian Hospitals, Gdynia, Poland; 3 Oncologic Hospital, Tomaszów Mazowiecki, Poland; 4 Department of Oncology, Oncology Centre, Bydgoszcz, Poland; 5 Department of Hematology, Oncology and Internal Diseases, Medical University of Warsaw, Warsaw, Poland; 6 Department of Lymphoid Malignancies, Maria Sklodowska-Curie National Research Institute of Oncology, Warsaw branch, Warsaw, Poland; 7 Department of Hematology, Independent Public Health Care Ministry of the Interior of Warmia and Mazury Oncology Center, Olsztyn, Poland; 8 Department of Hematology and Transplantology, Medical University of Gdańsk, Gdańsk, Poland; 9 Department of Hematology, Blood Neoplasms and Bone Marrow Transplantation, Wrocław Medical University, Wrocław, Poland; 10 Department of Hematology, Jagiellonian University, Krakow, Poland; 11 InternalMedicine and Oncology Clinic, Silesian Medical University, Katowice, Poland; 12 Department of Hematology, Military Institute of Medicine, Warsaw, Poland; 13 Department of Hematology, Ludwik Rydygier Hospital, Krakow, Poland; 14 Department of Biostatistics and Bioinformatics, Maria Sklodowska-Curie National Research Institute of Oncology, Gliwice Branch, Gliwice, Poland

**Correction to: Annals of Hematology**

10.1007/s00277-021-04448-5

In the above-mentioned article, the affiliation of one author Magdalena Olszewska-Szopa is not correct. It is presented as 8 (Department of Hematology and Transplantology, Medicl University of Gdańsk). It should be 9 (Department of Hematology, Blood Neoplasms and Bone Marrow Transplantation, Wrocław Medical University, Wrocław, Poland).

The original article has been corrected.

